# The first national public breast cancer screening program in Saudi Arabia

**DOI:** 10.4103/0256-4947.67078

**Published:** 2010

**Authors:** Omalkhair A. Abulkhair, Fatina M. Al Tahan, Susan E. Young, Salma MA. Musaad, Abdul-Rahman M. Jazieh

**Affiliations:** a From the King Saud bin Abdulaziz University for Health Sciences, Riyadh, Saudi Arabia; b Kendle International, Cincinnati, Ohio, United States

## Abstract

**BACKGROUND AND OBJECTIVES:**

Despite its relatively low incidence in Saudi Arabia, breast cancer has been the most common cancer among Saudi females for the past 12 consecutive years. The objective of this study was to report the results of the first national public breast cancer screening program in Saudi Arabia.

**METHODS:**

Women 40 years of age or older underwent breast cancer screening. Mammograms were scored using the Breast Imaging-Reporting and Data System (BI-RADS). Correlations between imaging findings, risk factors and pathological findings were analyzed.

**RESULTS:**

Between September 2007 and April 2008, 1215 women were enrolled. The median age was 45 years, and median body mass index was 31.6 kg/m^2^. Sixteen cases of cancer were diagnosed. No cancer was diagnosed in 942 women with R1/R2 scores, and only 1 case of cancer was diagnosed in 228 women with R0/R3 scores. However, among 26 women with R4/R5 scores, 50% had malignant disease and 35% had benign lesions. No correlation was found between known risk factors and imaging score or cancer diagnosis.

**CONCLUSIONS:**

Public acceptance of the breast cancer screening program was encouraging. Longitudinal follow-up will help in better determining the risk factors relevant to our patient population.

Breast cancer is the most common cancer worldwide. Incidence and mortality have reached a plateau and appear to be dropping in both United States and parts of western Europe.^1^,^2^ This decline has been attributed to several factors, such as early detection through the use of screening mammography and appropriate use of systemic adjuvant therapy.^3^

Breast cancer is influenced by multiple risk factors, which can be classified into 4 groups: first, family history/genetic background, which accounts for approximately 15% of all breast cancer cases.^4^ The second and the most well-known risk factor for breast cancer, can be linked to the hazardous effects of hormonal exposures such as early age at menarche, late age at menopause,^5^ fewer number of children and nulliparity, late age at first birth,^6^ little or no breastfeeding and long-term use of hormone replacement therapy (HRT).^7^–^12^ The third is high breast density, which has been shown to be one of the most significant markers of breast cancer risk;^13^ and the fourth, a history of benign proliferative breast disease.^14^

Recently, there is emerging evidence that overall caloric intake and obesity with weight gain in particular are related to increased breast cancer risk with different effects for premenopausal and postmenopausal women.^15^,^16^ Although these factors have been thoroughly studied and accurate quantitative estimates for risk are now available for the western population, there have been no studies for Arab women.

Despite the relatively low incidence of breast cancer in Saudi Arabia compared to other countries, it has been the most common cancer among Saudi females for the past 12 consecutive years (Saudi Cancer Registry, 1994-2005). Data on female patients with invasive breast carcinoma reported from different regions in Saudi Arabia show that most patients are in the age group of 40 to 50 years and were predominantly premenopausal. More than 50% were stage II and III, while ductal carcinoma in situ represented <5% of this population.^17^–^22^ Although breast cancer is more common in women older than 50 years worldwide, it is frequently diagnosed in younger women in Saudi Arabia. In fact, breast cancer is the single leading cause of cancer death for women 20 to 59 years of age,^23^ thus posing a major public health concern. The high incidence of breast cancer in young Saudi women should be addressed by evaluating the roles of early detection and prevention programs. In addition, correlation between common risk factors for breast cancer must be identified.

It is now well established that early detection provides survival advantages to women with breast cancer. Mammography, which detects breast cancer at earlier stages, is a major step in reducing the risk of death from this disease. It was estimated to prevent approximately 20% to 40% of all deaths from breast cancer among women undergoing screening mammography.^24^–^27^ In 1997, the first published results of the New York randomized controlled trial of breast cancer screening^28^ indicated that a program combining physical examination and mammography at annual intervals was successful in reducing breast cancer mortality in women aged 50 years and older. These findings were confirmed by the same researchers in a 14-year follow-up of the original study group and control population.^29^,^30^ Subsequently, a Swedish study^31^ showed that mammographic screening alone was capable of achieving a significant reduction in mortality. This finding was confirmed in two other trials in the Netherlands.^32^,^33^ Of the earlier studies, only one,^34^ the Health Insurance Plan (HIP) trial, has demonstrated the effectiveness of breast cancer screening in women aged 40 to 49 years and only after prolonged follow-up.

On the other hand, many women have no access to mammography due to racial, environmental, financial/ insurance barriers; lack of education; and, most importantly, lack of encouragement by a physician.^35^,^36^ In Saudi Arabia, the low utilization of mammography screening is mainly attributed to lack of education and awareness among females.^37^ Earlier studies conducted in different regions of Saudi Arabia, such as Al-Qaseem, Riyadh, Jeddah and Dammam, have explored female knowledge of, and attitude towards, breast cancer.^37^–^39^ Major factors identified included lack of knowledge about the common risk factors for breast cancer; and lack of understanding of the importance of breast self-examination, which is the best option for internal screening among women of all ages. Another important hindrance to tackling the problem of under-utilized mammography screening is the lack of standard national screening programs.^38^ In fact, a study concluded that the unavailability of a national screening program and the lack of women’s cooperation and trust were the main barriers to the implementation of screening by primary healthcare physicians (PHCPs).^39^

Considering the growth and aging of the population in Saudi Arabia, cancer rates are expected to increase considerably. This will add an enormous burden to the healthcare-utilization costs.^21^ The objectives of this study were to describe the results of the first national public breast cancer screening program in Riyadh, the capital city of Saudi Arabia, and to evaluate the relationship among common risk factors and the relationship of common risk factors with BI-RADS score and with breast cancer diagnosis in this Saudi population.

## METHODS

### Establishing the center

The first nationwide breast cancer screening center started accepted participants from all regions, but the majority came from Riyadh since that was the location of the center. The Abdul Lateef Charitable Screening Center was established in Riyadh by a generous donation from a prominent businessman (Shaikh Abdul Lateef Mohamed Abdul Lateef). Screening commenced in September 2007, but the official inauguration of the center took place on October 23, 2007, and was graced by the presence of Mrs. Laura Bush, the former first lady of the United States and a representative from the Susan G. Komen for Cure Organization, who visited the center as part of their tour to the Middle East to promote cancer awareness.

In partnership with the Saudi Cancer Society, a well-designed public-awareness program was launched year-round, and women were encouraged to visit the center to be screened for breast cancer. Attendants at the center were directed to a female-exclusive area, wherein a trained female technician, a family physician and a health educator were available. All women who visited the center were self-referred.

### Study design

This was a retrospective study conducted on patients attending the screening center. The study was approved by the Research Committee and Institution Review Board (IRB) of the King Abdullah International Medical Research Center at King Abdulaziz Medical City (KAMC). Data on participants in this study are stored at the screening center, as well as in the KAMC.

### Data collection and analysis

This was a population-based breast cancer screening registry designed to collect extensive information on known and potential breast cancer risk factors in all women who visit the center.

The main objective of this preliminary analysis was to assess the prevalence of breast cancer phenotypes in Saudi women. Additional objectives were to evaluate the association of a diagnosis of breast cancer with the BI-RADS score, potential risk factors and the acceptability of breast cancer screening in this population.

The data collected from the screened participants included the following well-known risk factors for breast cancer: age, hormonal status, early menarche, menopausal status, late parity and nulliparity, HRT and breastfeeding status, previous breast pathology and family history.

The only inclusion criterion for the current study was being an asymptomatic woman of age ≥40 years; however, since we were providing the first breast cancer screening in our local community, we included some women with symptoms to provide as much population-based data as possible in order not to deprive these women from access to this potentially life-saving test.

Conventional 2-view film/screen mammograms were provided and reviewed by two radiologists. Further views were performed when judged to be necessary. Mammograms were scored on a 5-point scale using the American College of Radiology Breast Imaging-Reporting and Data System (BI-RADS). The categories are shown in Table 1.^40^ Based on mammography results, women who needed further investigations were referred to KAMC for further workup and management.

**Table 1:** Breast Imaging-Reporting and Database System (BI-RADS).

Category	Assessment	Follow-up
0	Need additional imaging evaluation	Additional imaging needed before a category can be assigned
1	Negative	Continue annual screening mammography (for women over age 40)
2	Benign (non-cancerous) finding	Continue annual screening mammography (for women over age 40)
3	Probably benign	Receive a 6-month follow-up mammogram
4	Suspicious abnormality	May require biopsy
5	Highly suggestive of malignancy (cancer)	Requires biopsy
6	Known biopsy—proven malignancy (cancer)	Biopsy confirms presence of cancer before treatment begins

Adopted from: http://www.cancer.gov/cancertopics/factsheet/Detection/screening-mammograms

### Main outcome variable, definitions and tests

The main outcome variable was a diagnosis of breast cancer. A breast cancer diagnosis was determined according to the results of fine-needle aspiration and/or biopsy with a written pathological report.

Risk factors were patient-reported unless otherwise stated and were defined as follows:

- A positive family history: the presence of a diagnosis of breast cancer in one or more of the patient’s direct blood relatives (mother, sister and daughter)
- Estrogen use (hormone replacement, contraceptive pills): a yes response to “ever used estrogen, currently use estrogen or both”
- Early menarche: women who started their period before 12 years of age
- Menopausal status: women who had menopause at the time of screening, defined as “those who reported an age at which menopause started”
- Late parity: women who had their first child after the age of 30 years (women with nulliparity were defined as women who did not have any children or who did not report any pregnancies [term or non-term] at the time of the study)
- Previous breast surgeries included mastectomy, fine-needle aspiration, breast reconstruction (implants) and other surgeries

Patients were symptomatic if they had a history of breast mass, nipple inversion or retraction, pain, nipple discharge or skin changes on clinical examination. Patients were asymptomatic if they lacked all these symptoms. The BI-RADS score was defined as shown in the data collection section. If a patient had a second BI-RADS assessment conducted after the first, the second BIRADS score was utilized in the analysis. Follow-up tests consisted of imaging or procedures performed after the last BI-RADS assessment. Follow-up imaging tests included one or more of the following: mammogram, ultrasound or MRI. Follow-up procedures consisted of fine-needle aspiration, biopsy or both.

### Statistical methods

Demographic and baseline characteristics (age, body mass index [BMI], risk factors) were descriptively summarized. The descriptive statistics for continuous variables were mean and standard deviation, median and minimum and maximum. Frequency counts and percentages were tabulated for categorical variables. Categorical data was assessed using the chi-square or the Fisher exact test. Two-sided tests at the 5% significance level were utilized.

Logistic regression was used to explore the relationship of a malignant cancer diagnosis as the binary outcome variable with the risk factors (family history, estrogen use [hormone replacement], early menarche, menopausal status, late parity, nulliparity, previous breast surgeries or biopsy) and the BI-RADS score. In the logistic regression, variables were selected using backward selection at the 10% significance level in this preliminary analysis. Data was analyzed using SAS version 9.1.2 (SAS Institute Inc., Cary, NC, USA).

## RESULTS

Between September 2007 and April 2008, 1215 women were enrolled in the program (Table 2). The majority of screened women were from Riyadh. The median age was 45 years (range, 19-91), and the median BMI was 31.6 kg/m2 (range, 16.7-58). The mean (SD) age was 46.5 (8.1) years and the mean (SD) BMI was 32.0 (5.6) kg/m ^2^. Risk factors among participants included a positive family history in 11.7%, early menarche in 11.9%, and menopause in 24.2%; only 9.3% had late parity and 4.4% were nulliparous. Approximately 5.6% of the women had a history of previous breast biopsy or fine-needle aspiration (FNA), reported as a benign lesion. The majority were asymptomatic (n=607), while 475 were symptomatic. Breast pain was the most common symptom reported (n=379) (Table 2).

**Table 2:** Characteristics of women enrolled in the screening program (n=1215).

	n (%)
Family history cancer	
Positive family history	142 (11.7)
Negative family history	1064(87.6)
Unknown or missing	9 (0.7)
Estrogen use	
Yes	874 (71.9)
No	202 (16.)
Unknown or missing	139 (11.4)
Early menarche	
Yes	145 (11.9)
No	1042 (85.8)
Unknown or missing	28 (2.3)
Menopausal status	
Yes	294 (24.2)
No	883 (72.7)
Unknown or missing	38 (3.1)
Late parity	
Yes	113 (9.3)
No	1073 (88.3)
Unknown or missing	29 (2.4)
Nulliparity	
Yes	53 (4.4)
No	1145 (94.2)
Unknown or missing	17 (1.4)
Previous breast surgery or biopsy	
Yes	68 (5.6)
No	1142 (93.9)
Unknown or missing	5 (0.4)
Symptoms	
Symptomatic^a^	475 (39.1)
Breast mass	183 (15.1)
Nipple inversion or retraction	22 (1.8)
Breast pain	379 (31.2)
Nipple discharge	16 (1.3)
Asymptomatic	607 (50.0)
Unknown or missing	133 (10.9)

a Raw numbers of the individual symptoms do not add up to the total due to overlap (one patient may have more than one symptom). Percentages of the individual symptoms are calculated from the total sample (n=1215).

For the BI-RADS scores, 54.5% of all the women were R1, 23.2% were R2, 9.2% were R3, 1.3% were R4, 0.8% were R5 and 9.6% were R0. Table 3 presents the mammographic screening scores based on BI-RADS and cancer diagnosis. The BI-RADS scores were combined as R1R2, R3R0 or R4R5. The rationale for combining the scores into R1R2 is that they were considered benign and no further tests were needed. For R3R0, the rationale was that these categories were subject to another imaging study. For R4R5, the rationale was that the suspicion of malignancy was the highest, requiring these women to undergo other tests. Of 942 women with an R1R2 BI-RADS score, 10.3% underwent follow-up imaging because of high breast density, while none underwent a follow-up procedure. Of 229 women with an R3R0 BI-RADS score, 26.2% underwent follow-up imaging, while only 0.9% underwent a follow-up procedure. A small proportion of the total population of women had a combined BI-RADS score of R4R5 (n=27); as expected, a large proportion of these women had follow-up imaging (62.9%) and a follow-up procedure (51.9%). Among the patients with an R3R0 diagnosis, only 2 (0.9%) had a confirmed malignant tumor, while a considerably higher proportion (20.1%) had a confirmed benign tumor. In contrast, about half (51.8%) of the women with an R4R5 score had a confirmed malignant tumor, while 33.3% had a benign tumor. Those who were reported R1/R2 had no further studies apart from their annual screening.

**Table 3:** Summary of the screening mammography findings (n=1215). Percentages of the follow up tests and cancer diagnoses are based on the number of subjects in each BI-RADS classification group.

BI-RADS	n (% of total population)	F/U Imaging*	F/U Procedure*	Number of cancer cases with confirmed diagnosis	
				Malignant*	Benign*
R_1_ R_2_	942 (77.5)	0	0	0	0
R_3_ R_0_^a^	229(18.9)	60 (26.2)	2 (0.9)	2 (0.9)	46 (20.1)
R_4_ R_5_	27 (2.2)	17 (62.9)	14 (51.9)	14 (51.8)	9 (33.4)
No mammogram^b^	17 (1.4)	1 (5.9)	0	0	1 (5.9)F

F/U: follow up.

* *P*<.0001 using Fisher exact test

a Some subjects with a combined BIRADS score of R3R0 have not been included in the results as at the close of the study, they had refused follow up or insufficient time had elapsed for the follow up imaging to be performed.

b 17 subjects did not have initial screening mammogram due to either young age, fear of procedure or non-availability of technician at the time of the screening visit.

The histological findings for the benign and malignant lesions identified to date in this population are summarized in Table 4. Of a total of 83 patients with a histological diagnosis, the most frequent benign lesions identified were cysts (22.9%), followed by mammary dysplasia (12.0%). Malignant lesions consisted of infiltrating ductal carcinoma, identified in 15 (18.1%) patients, and ductal carcinoma in situ, identified in 1 (1.2%) patient. The proportion of patients with a malignant breast cancer diagnosis proportionately and significantly increased with the BI-RADS score (P< .0001) (Table 5). Among the 27 patients with an R4R5 BI-RADS score, 14 had a confirmed malignancy (4 with R4 and 10 with R5). Two patients with an R3 classification were diagnosed with cancer.

**Table 4:** Description of pathological diagnosis of abnormal breast imaging findings (n=83).

Histological diagnosis	n (%)
Benign lesions	
Cyst	19 (22.9)
Benign mammary dysplasia, unspecified	10 (12.0)
Mammographic microcalcification	7 (8.4)
Other abnormal findings on radiological exam	6 (7.2)
Fibroadenosis	6 (7.2)
Benign neoplasm of breast	5 (6.0)
Other specified benign mammary dysplasias	7 (8.4)
Mammary duct ectasia	3 (3.6)
Inflammatory disease	2 (2.4)
Galactocele	1 (1.2)
Accessory breast	1 (1.2)
Diffuse cystic mastopathy	0
Fibrosclerosis	0
Abnormal mammogram, unspecified	0
Malignant lesions	
Infiltrating ductal carcinoma	15 (18.1)
Ductal carcinoma in situ, solid type	1 (1.2)

**Table 5:** The association of BI-RADS score with a malignant breast cancer diagnosis using logistic regression (n=1215). Percentages of malignant breast cancer were calculated from the BIRADS classification group in each row.

BI-RADS classification	n (% of total population)	Confirmed malignant breast cancer n (%)*
R_1_	661 (54.4)	0
R_2_	281 (23.1)	0
R_3_	113 (9.3)	2 (1.8)
R_4_	16 (1.3)	4 (25.0)
R_5_	11 (0.9)	10 (90.9)
R_0_	116 (9.6)	0

* *P<*.0001 using Fisher exact test. Note: 17 subjects did not have initial screening mammogram due to either young age, fear of procedure or inabilitity to schedule screening test.

Logistic regression was utilized to model the relationship of the outcome of a malignant breast cancer with the risk factors and the BI-RADS score. Results revealed a significant relationship of BI-RADS score with the outcome. Women with an increasing BI-RADS score were approximately two times more likely to have malignant breast cancer (odds ratio=1.96; 95% confidence interval=1.5-2.6) (*P*<.0001). None of the other risk factors were significantly associated with the outcome (not shown). The percentage of localized breast cancers detected was found to be double that of the Saudi National Cancer Registry data (50% vs. 25.5%, respectively) (Table 6).

**Table 6:** Stage at diagnosis.

	Current study (n%)	Saudi Arabia, 2005^a^n (%)
Stage		
In situ	1 (6.3)	n/a
0	1 (6.3)	
Localized	8 (50.0)	(25.4)
I	2 (12.5)	
IA	1 (6.3)	
IIA	5 (31.3)	
Regional	3 (18.8)	(44.6)
IIB	3 (18.8)	
Distant	3 (18.8)	(12.4)
IV	3 (18.8)	
Unknown	1 (6.3)	(17.5)
Total	16 (100)	932 (100)

a Cancer Incidence Report, Saudi Arabia 2005, Saudi Cancer Registry

## DISCUSSION

The current report is the first of a population-based breast cancer screening program of its kind in Saudi Arabia. This study aimed at presenting an overview of the acceptance level of Saudi women for breast cancer screening and evaluating the relationship between common risk factors and breast cancer in Saudi population, in addition to evaluating the association between BI-RADS score and breast cancer diagnosis.

Approximately 10 years ago, the WHO predicted that an increase in life expectancy^41^ and drastic changes in life style are expected to lead to an epidemic of breast cancer in the majority of developing countries by the first quarter of the next century. In line with this prediction, it is estimated that 70% of the new cases of cancer, including breast cancer, will be diagnosed in people living in developing countries by the year 2020.^42^

In a recent publication, Ibrahim et al estimated that the future burden of breast cancer in Saudi Arabia is expected to increase by approximately 350% by 2025.^21^ The available data in Saudi Arabia is predominantly related to awareness and perceptions of women and health professionals about screening, as well as their attitude towards it,^24^,^39^ but there is no data on actual screening programs. The only available screening program is established in Al-Qaseem, Saudi Arabia, run by the local health department in cooperation with the King Abdulaziz Women’s Charity Committee. This program employed a mobile mammography van to reach remote areas in the province. Results of this ongoing screening program are not yet available; however, they will be important as the program is the first in the world to screen a population under the age of 40.^43^

What we can learn from this program so far is that with well-conducted breast cancer awareness programs, women themselves will be encouraged to come for screening. Another pilot study was conducted by our group in Riyadh in 2006 as an outreach program. Our main conclusion was the dire need for health education and constant awareness campaigns, in addition to making the test accessible.^44^ The involvement of primary healthcare physicians is very important for the success of the screening program.

In addition, our observations during the visit to the country by U.S. former first lady Laura Bush, along with all of the accompanying media coverage, highlight the importance of the role of media in the advertising/ awareness campaigns. After the inauguration of the center till the arrival of Mrs. Bush, a few women per day were being screened at the center; however, after Mrs. Bush’s visit, those numbers increased noticeably. The use of media is especially important in our population due to the high percentage of illiteracy.

Finally, the strong correlation between mammographic findings and breast cancer confirmation is an encouraging finding for the center. This means that mammogram is an effective tool in detecting breast cancer in our patient population, which is generally young and may have denser breast tissue compared to elderly women. Long-term follow-up is required to assess the actual benefit; however, results are promising. Out of 16 confirmed malignancies, 8 cases were localized disease and 3 were regional disease. It is noteworthy that the detection rate of localized stages of breast cancer is double the rate revealed on the basis of the National Cancer Registry data. Although the sample size was small, the study is encouraging as it authenticates the goal of screening programs, which is to detect cancer at earlier, potentially more curable stages. There was also one case of ductal carcinoma in situ. Long-term follow-up is definitely needed to assess the impact on outcome. In addition, diagnosis of ductal carcinoma in situ and Stage I disease is an encouraging sign since diagnosis of disease at a lower stage will have a significant effect on outcomes.

Our study has a few limitations, including the fact that we had to include women with symptoms, which may contaminate what is supposed to be a “pure screening study.” However, our concerns about the well-being of our patients made us accommodate these patients who may not otherwise have had their cancer detected at an earlier stage for many reasons, including lack of access to care, among other aforementioned barriers.

This issue will face any pioneer programs in areas where new tests are being introduced and people find easy access to address their concerns through such programs. The fact that many women with symptoms came to the center raises concerns about the available options for these women outside the center and requires further evaluation. The study did not confirm the value of the well-established and known risk factors of breast cancer. It is too early to accept this as a matter of fact, and analysis of a larger cohort or longer follow-up or a different study design may be needed to address this issue.

In conclusion, breast cancer screening is acceptable to our female population, which responded to the media campaign. Using BI-RADS was helpful in identifying malignant lesions with high accuracy. Determining breast cancer risk factors requires further investigations.
